# Multiport Combined Endoscopic Approach to Nonembolized Juvenile Nasopharyngeal Angiofibroma with Parapharyngeal Extension: An Emerging Concept

**DOI:** 10.1155/2016/4203160

**Published:** 2016-12-22

**Authors:** Tiruchy Narayanan Janakiram, Shilpee Bhatia Sharma, Vijayshree Nahata Gattani

**Affiliations:** Royal Pearl Hospital, Tiruchirappalli, Tamil Nadu, India

## Abstract

*Background*. Surgical approaches to the parapharyngeal space (PPS) are challenging by virtue of deep location and neurovascular content. Juvenile Nasopharyngeal Angiofibroma (JNA) is a formidable hypervascular tumor that involves multiple compartments with increase in size. In tumors with extension to parapharyngeal space, the endonasal approach was observed to be inadequate. Combined Endoscopic Endonasal Approaches and Endoscopic Transoral Surgery (EEA-ETOS) approach has provided a customized alternative of multicorridor approach to access JNA for its safe and efficient resection.* Methods*. The study demonstrates a case series of patients of JNA with prestyloid parapharyngeal space extension operated by endoscopic endonasal and endoscopic transoral approach for tumor excision.* Results*. The multiport EEA-ETOS approach was used to provide wide exposure to access JNA in parapharyngeal space. No major complications were observed. No conversion to external approach was required. Postoperative morbidity was low and postoperative scans showed no residual tumor. A one-year follow-up was maintained and there was no evidence of disease recurrence.* Conclusion*. Although preliminary, our experience demonstrates safety and efficacy of multiport approach in providing access to multiple compartments, facilitating total excision of JNA in selected cases.

## 1. Introduction

JNA is a hypervascular benign neoplasm known for its peculiar local aggressive spread along the pathways of least resistance. This tumor is of considerable scientific interest due to its vascularity and high incidence of recurrence. Numerous open surgical approaches have been employed traditionally, based on the tumor extensions, like transpalatine, lateral rhinotomy, midfacial degloving, and neurosurgical approaches in cases with intracranial extension [1]. The multiplicity of approaches developed over the years corroborates with the inaccessibility of the lesion during surgical resection.

The adoption of endoscopes in surgery for the resection of JNA came in vogue in the late 1990 and thereafter evolved rapidly [2]. The advent of minimally invasive approaches has significantly altered the realm of skull base surgery for management of JNA. Technological advancements, new corridors, and increased surgical experience have made endoscopic resection the modality of choice for small to medium size JNA [3, 4]. Principles for successful total endoscopic resection of JNA have evolved over the years allowing authors to consider endoscopy as the choice of approach for advanced tumors. The principles of wide exposure, dissection along tumor bed and four-handed binostril technique have allowed for successful resection of advanced tumors [2].

The parapharyngeal extension is a surgical challenge due to its vicinity to neurovascular structures. The endonasal approach was observed to be inadequate for total control of the inferolateral part of the lesion [5]. Thus, compelling the surgeon to resort to the traditional open approaches to address tumors occupying the parapharyngeal space. These approaches were associated with high morbidity, blood loss, and scarring [6].

Ehrlich first described the transoral approach to the parapharyngeal space in 1950 but concluded to discourage this approach due to complications [7]. In 2010, Lee et al. published the first endoscopic PPS approach for draining a pediatric abscess [8]. Subsequently, many authors published reports on transoral and transcervical endoscopic approaches for benign PPS tumors [9, 10]. In recent years pioneering work has been done by many authors to contribute to better understanding of the complex three-dimensional anatomy by employing EEA with TORS [11–14].

In this study we present our experience of two cases of JNA with prestyloid parapharyngeal space involvement, surgically managed by an alternative approach to the parapharyngeal space, a combined endoscopic endonasal approach (EEA), and endoscopic transoral approach (ETOS) ensuring complete resection. The advantages and limitations of this technique were evaluated.

## 2. Materials and Methods

A retrospective analysis of two patients of JNA operated on in 2015 at our center by combined multiport EEA and ETOS was conducted. Local ethical committee approved the study. Any extension to the poststyloid compartment of the parapharyngeal space was excluded from the study.

The patients were subjected to a thorough preoperative workup, which included history taking and detailed examination including cranial nerve function, endoscopic examination, and Contrast Enhanced Computed Tomography (CECT) of paranasal sinus and oral cavity. The CECT was analyzed closely for the extent of tumor, parapharyngeal compartment involvement, skull base erosion, and vicinity to neurovascular structures. A Digital Subtraction Angiography ruled out the presence of any feeders from the parapharyngeal part of internal carotid artery or any aberrant anastomosis. No embolization procedures were done in any of the patients.

The patients were fully informed about the details of the planned surgical procedure, complications, and chances of resorting to an open procedure in case of inadequate control. They were also counseled about the advantages and drawbacks of the possible alternative therapies. A written informed consent was obtained from all the patients. A single experienced skull base surgeon performed surgical procedures.

Postoperatively the hospital stay was around 1 week. Prophylactic IV antibiotics were administered and nasogastric feeding was initiated to allow faster healing of oropharyngeal wounds. Special attention was paid towards maintaining optimal oral hygiene, apart from routine postoperative care. Nasal packing was removed after 36 hours and saline douching was advised. A surveillance CECT scan was done after 36–48 hours to confirm no residual tumor. A monthly follow-up with endoscopic nasal and oral examination was done for two months and then every six months. The surveillance scan was done after one year to evaluate for any recurrence.

### 2.1. Surgical Technique

The surgery was performed under controlled hypotension using four-handed binostril endoscopic technique. Navigation system was installed for anatomical orientation during the surgery. Rod lens endoscopes (4 mm diameter, 18 cm length) with 0-degree lenses coupled to a high-definition camera and monitor (SPIES™, Karl Storz, Tuttlingen, Germany) gave excellent visualization during the surgery. The control of the external carotid artery was performed in the neck on the same side by ligating it to devascularize the tumor.

On the basis of tumor extension an intraoperative surgical trajectory was selected to provide maximal exposure with minimal invasiveness. The principle of centripetal approach was employed for complete exposure of the tumor before resection. The nasal part of the tumor was debulked by segmental and piecemeal resection to increase access and surgical maneuverability in mobilizing the distal part of the tumor. Four-handed technique ensured good visibility of surgical field and identification of the tumor planes for dissection.

The surgical procedure can be divided into 3 steps, tailored according to the tumor extensions.

*(1) Transnasal Approach*. Modified endoscopic Denker's procedure was performed to provide adequate exposure. Using a knife, under endoscopic guidance, an incision was placed on the anterior edge of the pyriform aperture. Freer's elevator was then used to elevate a flap in the subperiosteal plane to expose the anterolateral wall of the maxilla. The superior limit of the exposure was up to the infraorbital nerve. Care was taken not to injure the nerve during the dissection. Using a 4 mm cutting burr the anterolateral wall of the maxillary sinus was drilled. The nasolacrimal duct was exposed and transected. The exposure of the bone medially was done posteriorly till the level of the junction of the palatine bone and the medial pterygoid plate in which the descending palatine neurovascular bundle was located and cauterized. Laterally posterior wall of maxilla is removed till the lateral most extend of the tumor. Posterior septectomy was performed. The tumor in the nasal cavity was debulked to increase space for instrumentation. Bilateral ethmoidectomy and sphenoidotomy was done using a microdebrider to achieve control of the posterior most extent of the tumor.

**Figure 1:**
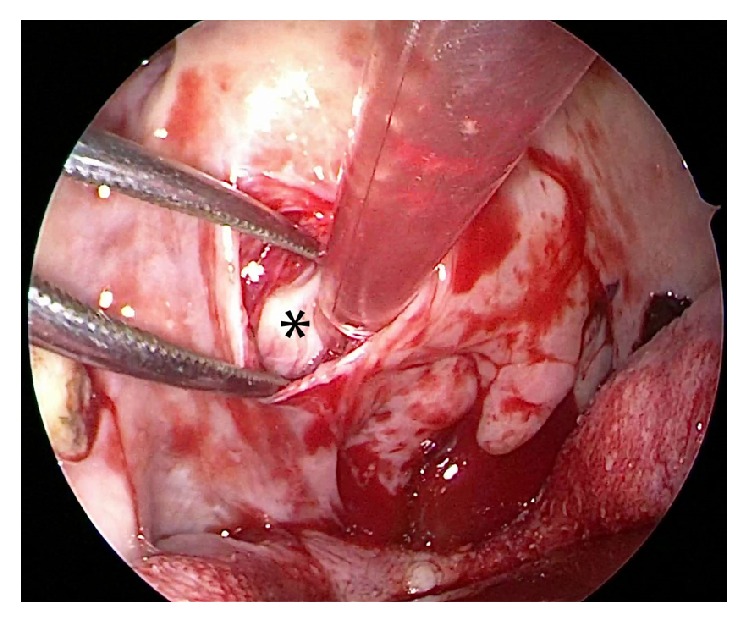
Endoscopic view showing the tumor (*∗*) which is exposed after giving submucosal incision and dissecting the fibers of superior constrictor.

**Figure 2:**
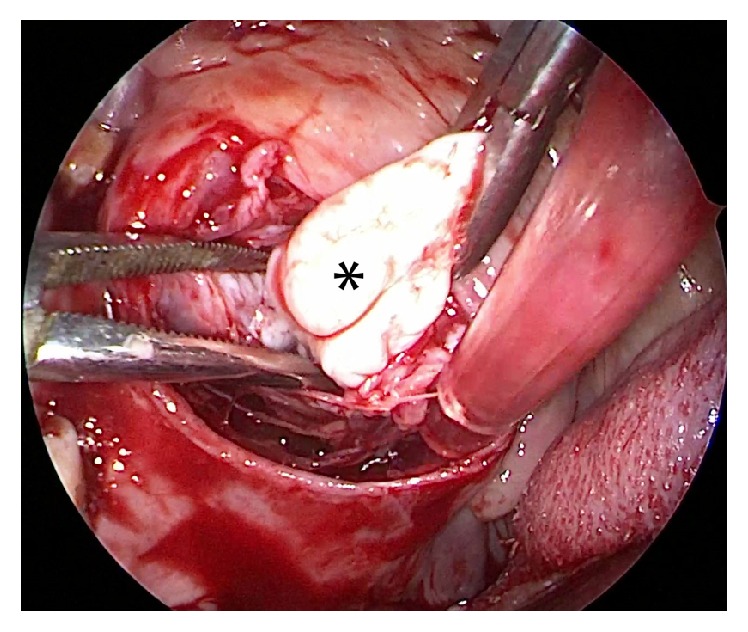
Endoscopic view showing the tumor (*∗*) being dissected with constant transaction.

**Figure 3:**
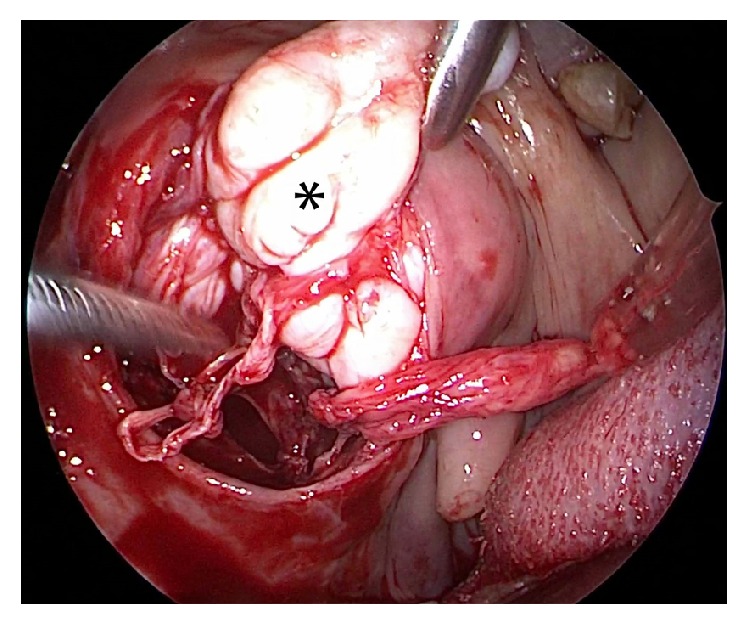
Endoscopic view showing the tumor (*∗*) being dissected out bluntly from the prestyloid space.

**Figure 4:**
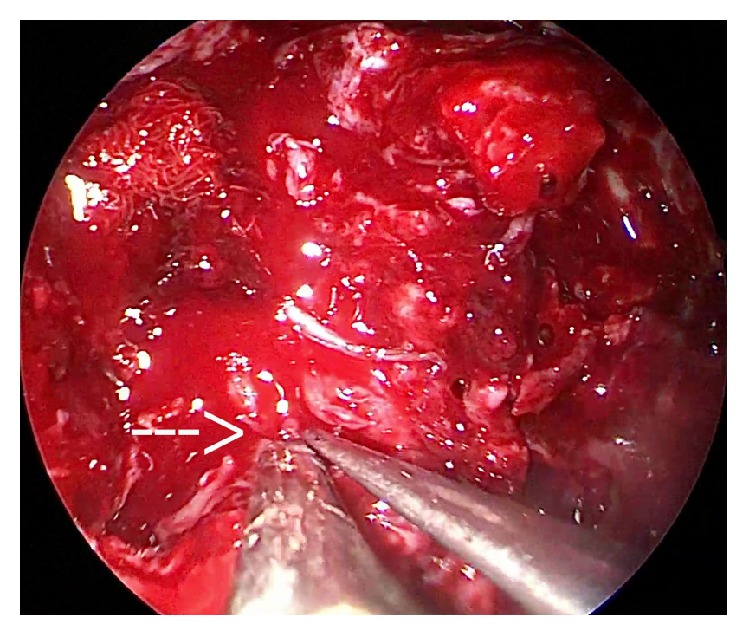
Endoscopic view of tumor being dissected out at V3 as illustrated by broken white arrow.

**Figure 5:**
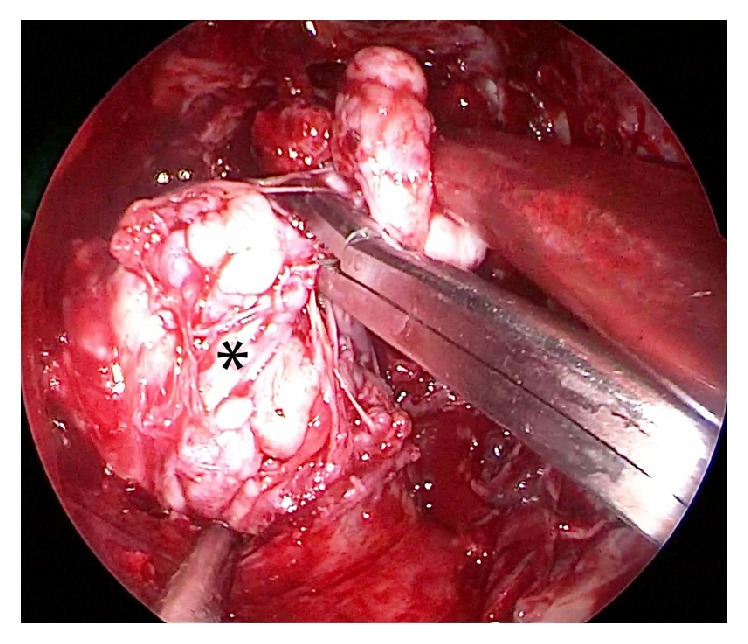
Endoscopic view showing the tumor (*∗*) being pushed superiorly.

*(2) Transoral Approach*. A Boyle Davis mouth gag was inserted to expose the soft palate. The tumor bulge was identified and an incision was placed over it. The mucosa, submucosa, and fibers of superior constrictors were dissected to expose and visualize the tumor (Figure 1). The tumor was seen occupying the upper part of the prestyloid compartment in parapharyngeal space with medial pterygoid laterally and superior constrictor muscle medially. Constant traction on the tumor was kept and endoscope provided magnified view of the tumor margins and fibrous adhesions at tumor bed (Figure 2). With constant outward traction on tumor's inferior end, the cleavage plane was identified and the tumor was bluntly dissected from all its attachments in the prestyloid space (Figure 3). The tumor is pushed superiorly into infratemporal fossa. The attachment of the parapharyngeal tumor to the infratemporal tumor was cut near the foramen ovale (Figures 4 and 5). The intraoral part of JNA was delivered from the nasal cavity. Hemostasis was achieved and watertight sutures were used to close the incision.

*(3) Transpterygoid Approach*. The tumor in the infratemporal and sphenoidal part was mobilized. Posteriorly its attachment to buccopharyngeal fascia in nasopharynx was dissected. The tumor was debulked in front of the pterygoid wedge to get access to the cancellous bone of the greater wing of sphenoid. The pterygoid wedge was drilled using a diamond burr and the residual tumor was cleared until the vidian nerve was identified. The clinical report of each case is described in detail.

### 2.2. Case  1

**Figure 6:**
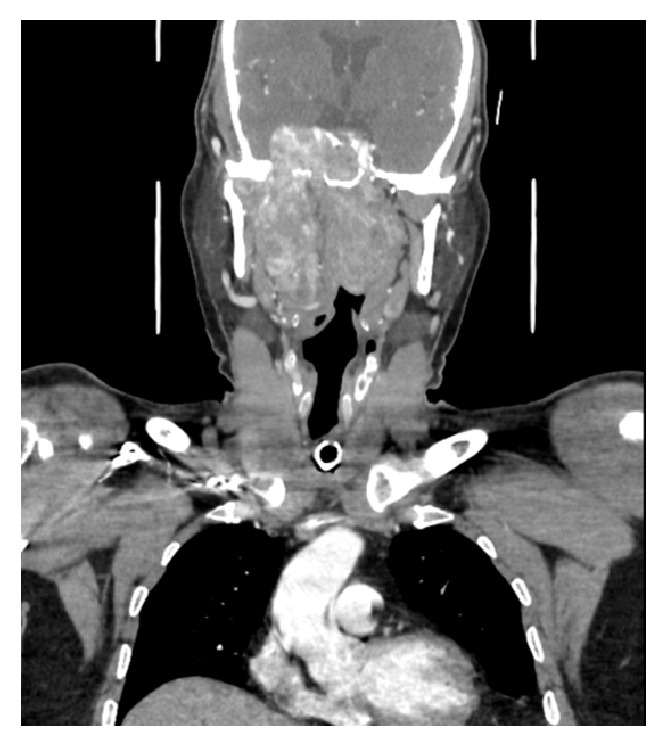
Coronal CT section (after contrast), showing an enhancing mass occupying the nasal cavity, sphenoid sinus, and infratemporal fossa and extending into the parapharyngeal space.

**Figure 7:**
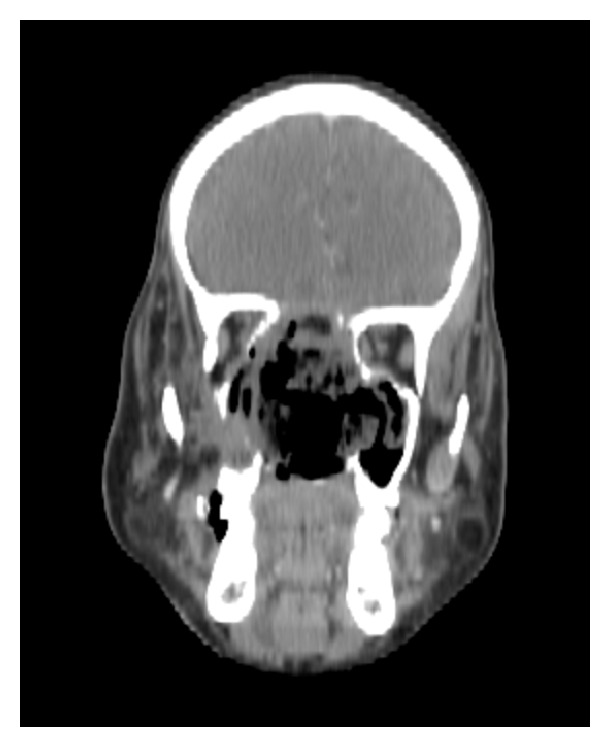
Coronal cut of contrast enhanced CT PNS showing postoperative changes and no recurrence.

A 21-year-old male patient presented to our Out Patient Department (OPD) with complaints of right sided nasal obstruction and intermittent epistaxis of 3 years duration. Endoscopic examination revealed a smooth fleshy mass occupying the right nasal cavity extending up to the external nares. A CECT scan of the paranasal sinuses revealed a homogenous mass, contrast enhancing, occupying the right nasal cavity, sphenoid sinus, pterygopalatine, and infratemporal fossa with deep extensions into the prestyloid upper parapharyngeal space laterally and skull base superiorly (Figure 6). Surgery was planned by a combination of EEA and ETOS. Complete tumor resection was achieved. Intraoperative time was around 5 hours with a blood loss of 2850 ml. Total of 4 units of blood transfusion was given. Histopathology report confirmed Juvenile Nasopharyngeal Angiofibroma. Intraoperative and postoperative periods were uneventful. No residual or recurrence was encountered on surveillance scans in follow-up (Figure 7).

### 2.3. Case  2

**Figure 8:**
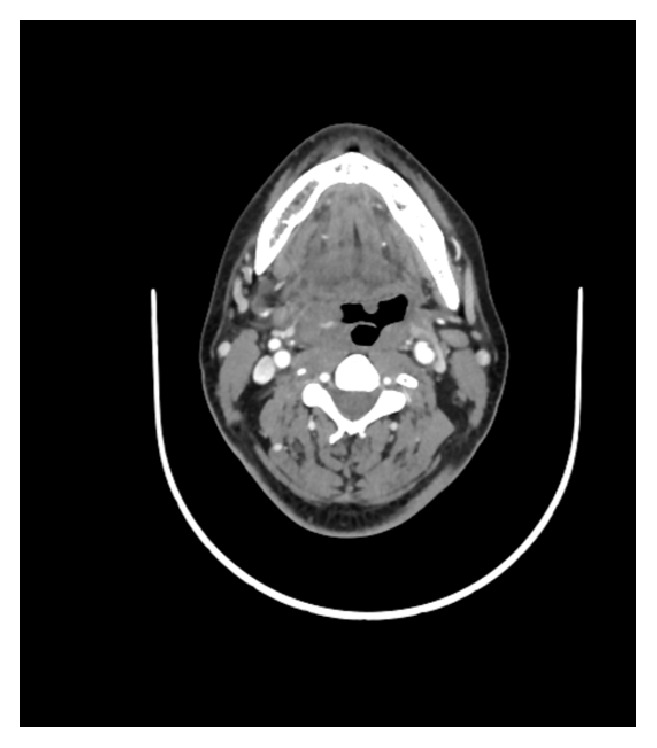
Axial cut of contrast enhanced CT scan of paranasal sinuses showing involvement of parapharyngeal space by Juvenile Nasopharyngeal Angiofibroma.

**Figure 9:**
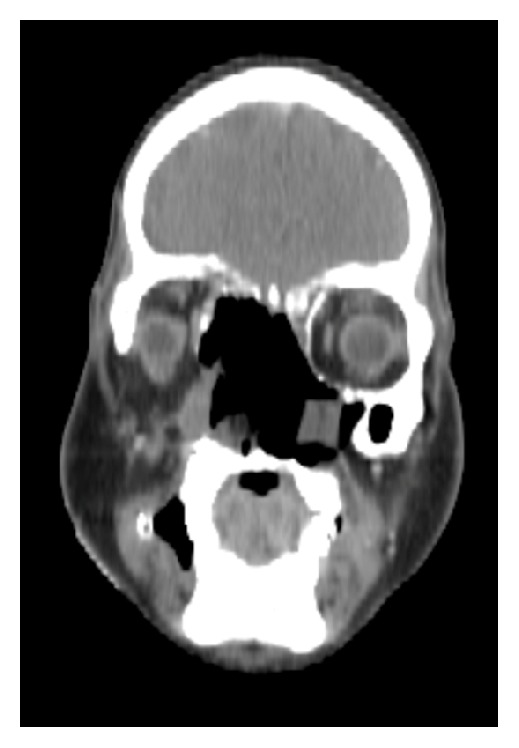
Coronal cut of contrast enhanced CT paranasal sinus showing postoperative changes and absence of any recurrence.

An 18-year-old male patient presented to our OPD with complaints of right sided progressive nasal obstruction and headache of 2-year duration. Endoscopic findings were consistent with that of a fleshy nasal mass in the right nasal cavity. MRI scan revealed an enhancing mass lesion in the right nasal cavity, pterygopalatine, and infratemporal fossa with lateral extension into the parapharyngeal space (Figure 8). The patient was taken for surgery by combined EEA and ETOS. Intraoperative period was uneventful. Blood loss of around 2675 ml was recorded. Patient received 3 whole blood transfusions and 1 packed cell transfusion. Histopathology report was consistent with Juvenile Nasopharyngeal Angiofibroma. Postoperatively the patient developed gaping of intraoral sutures and secondary resuturing was done. No residual or recurrences were encountered on surveillance scans (Figure 9).

Details of both cases have been summarized in Table 1.

## 3. Discussion

With the introduction of endoscopes, both purely endoscopic and endoscope-assisted resections of JNA have proved to be safe and efficient techniques [2]. The use of endoscopes in resection of JNA has slowly expanded from small and medium size to advanced stages of JNA [3]. Improved visualization with magnified, multiangled views and access to deep hidden areas has reduced risk of complications, leaving behind residual tumor [15, 16].

The tumor spreads along the paths of least resistance and extends into multiple compartments such as parapharyngeal and intracranial space. Most of these areas can be easily accessed by endoscopic approaches; however the surgical access to the inferolateral part of tumor in parapharyngeal space is beyond limits of endoscopic transnasal surgery, posing a surgical challenge. In such cases the surgeon often has to resort to open approaches.

Many surgical approaches to the parapharyngeal space have been described in literature; the most commonly employed are transcervical, transparotid, transmandibular, and infratemporal approaches or a combination of these [17]. However none of them completely fulfill the aim of total tumor removal with low morbidity, minimal scarring, and preservation of adjacent neurovascular structures [18]. Several important neurovascular structures in the parapharyngeal space narrow the surgical borders, making safe dissection difficult. Hence the surgical approach of choice should be as least traumatic, preserving the neurovascular structures, allowing adequate visualization and complete tumor removal [19].

Goodwin and Chandler first described the transoral approach to parapharyngeal space in 1988 [20]. The advantages such as avoidance of osteotomies and scars, no risk to facial nerve, and a short direct trajectory with least invasiveness were reported. However most authors did not advocate the use of this approach as a routine technique to parapharyngeal masses due to the limited exposure. They observed a higher risk of incomplete tumor removal, uncontrollable hemorrhage, and facial nerve injury due to its small blind access [21].

However along the years, advances in surgical techniques and instrumentation have made the transoral route an acceptable choice for small tumors in prestyloid compartment [22]. The increasing experience with endoscopic techniques has encouraged their use through new surgical corridors in approaching the deep compartments. Detailed anatomical studies based on cadaveric dissections improved understanding of parapharyngeal space anatomy and led to defining of landmarks for surgical orientation [23].

Dallan et al. first gave a step-by-step detailed description of the transoral access. They concluded that it is an excellent surgical window which provides great exposure for resection of parapharyngeal space lesions [24]. Turri-Zanoni et al. illustrated three cases of malignancy involving the prestyloid parapharyngeal compartments successfully operated using EEA-ETOS approach. They also noted that the involvement of middle cranial fossa, cavernous sinus, parotid gland, ramus of mandible, and engulfment of neurovascular structure in parapharyngeal space was contraindications for EEA-ETOS. They concluded that the combined EEA and ETOS approach to parapharyngeal space offered a better exposure and also reduced the postoperative morbidity when compared to open approaches [25].

Recently several authors have described Transoral Robotic Surgery (TORS) as a viable approach for resection of neoplasms of the parapharyngeal space with minimal morbidity. However this novel approach needs further evaluation [26]. The limitations of this technique include expensive and bulky instrumentation and lack of drilling equipment to manage skull base drilling [25].

In this study we applied these principles of EEA-ETOS to our carefully selected cases of JNA with parapharyngeal space involvement. We understand that this approach can be applied only with proper planning by experienced surgeons who can convert to an open approach when the need arises. We did not go for preoperative embolization as in our opinion it carries the risk of complications with increased procedural morbidity. It is also reported that surgical manipulation of tumor increasing chances of residual and recurrent disease [27]. Lloyd et al. and Li et al. have reported higher recurrences in these cases [27, 28]. In our practice, we devascularize the tumor by clamping the feeding vessel (internal maxillary artery or external carotid artery) of the same side thus avoiding any neurological sequelae. Although this procedure has risk of injury to surrounding structures or wound infection we still believe that advantage of complete resection outweighs the cons. Similarly the use of coblation or LASER for dissection and debulking was avoided in view of loss of tissue planes.

Two surgical trajectories provided best exposure to both nasal and parapharyngeal parts of the tumor. In the transnasal corridor, modified Denker's approach provided lateral access up to infratemporal fossa. Posterior septectomy and ethmosphenoidectomy gave access to nasopharyngeal and sphenoid part of tumor. We also routinely drill the pterygoid wedge to ensure complete removal.

The 4-handed technique with 2 experienced surgeons allowed for the use of four hands in the operating field, considerably improving visualization and reducing the surgical time. Endoscopes provided magnified view of the parapharyngeal space, allowing for safe dissection in correct plane under direct vision and preventing any unwanted injury to the surrounding neurovascular structures.

It was observed that a combined endonasal and transoral approach gave a good control of the tumor and allowed for complete resection under direct visualization. Also the total obviation of an open approach led to less blood loss, faster healing, and less morbidity and scarring. Avoidance of osteotomies in young patients helped in preventing disruption of the growing bony centers.

The rarity of JNA is evident from its low incidence of 0.4 cases per million and very few cases fit the inclusion criteria. The small number of patients, short follow-up, and the lack of control group are drawbacks of this study. The aim of our paper is to demonstrate use of multiport approach to enable individualized access to multiple compartments of JNA to facilitate its safe and efficient excision. The illumination and magnification provided by endoscopes render these techniques superior to external approaches in selected cases. This technique provides safe and effective alternative to both open approaches and TORS in selected cases.

## 4. Conclusion

The advent of endoscopic approaches for the skull base has paved the way for minimally invasive surgeries in complex lesions. Creating auxiliary surgical corridors can widen the limited trajectories to deep lateral areas. A total endoscopic approach has several obvious advantages over the open approaches that have been employed traditionally. The evolution of multiport approaches is a new dimension in management of complex skull base lesion with parapharyngeal extension. The concept of multiport surgery can provide a safe strategy for resection of selected case of benign and malignant lesions of skull base.

## Figures and Tables

**Table 1:** Summary of patient details.

Case	1	2
Age	21 years	18 years

Presentation	Right sided nasal obstruction and intermittent epistaxis since 3 years	Progressive right side nasal obstruction and headache since 2 years

Endoscopic findings	Smooth fleshy mass seen at right external nares	Fleshy mass in right nasal cavity

Radiological findings	Contrast enhancing mass occupying the right nasal cavity, sphenoid sinus, pterygopalatine, and infratemporal fossa with deep extensions into the prestyloid upper parapharyngeal space laterally and skull base superiorly	Enhancing mass lesion in the right nasal cavity, pterygopalatine, & infratemporal fossa with lateral extension into the parapharyngeal space

Surgical time	5 hours	4 hours

Blood loss		2675 ml

Surveillance		No residual/recurrence
